# Prospects and Applications of Biomass-Based Transparent Wood: An Architectural Glass Perspective

**DOI:** 10.3389/fchem.2021.747385

**Published:** 2021-10-20

**Authors:** Jing Wang, Jian’gang Zhu

**Affiliations:** College of Furnishings and Industrial Design, Nanjing Forestry University, Nanjing, China

**Keywords:** biomass materials, transparent materials, transparent wood, green and renewable energy, architectural glass;

## Abstract

This paper briefly discussed the research progress of biomass-based transparent wood (BBTW), and summarized the key technologies and potential application prospects of BBTW in replacing architectural glass. Based on the introduction of the preparation process of BBTW, the advantages of BBTW and their feasibility to replace architectural glass are illustrated with a view to the requirements and conditions of architectural glass for different use functions. The limitations of BBTW are discussed and the development prospects of BBTW are also prospected. The research shows that BBTW has the advantages of green and renewable materials that can meet the requirements of good lighting conditions, flame retardant, heat insulation and safety, which are in line with the sustainable development trend. Further studies are needed to continuously break through its limitations with an aim to expand the application of this new biomass-based material.

## Introduction

Biomass materials are new materials with excellent performance and high added value that are processed and manufactured by a series of high technology means using woody, grassy and vine plants (Sun and Liu, 2019; Wu et al., 2020; Zhao et al., 2020a; 2020b). Biomass materials are widely available and renewable, with low processing costs. In the current situation of depletion of non-renewable resources, the development and utilization of new materials can effectively alleviate the serious problems of energy tension and environmental pollution if renewable biomass materials are used as the “cornerstone” of new materials instead of traditional petrochemical and mineral-based materials (Liu et al., 2019; Wang et al., 2019; Chen L. et al., 2020).

Therefore, the research of biomass materials has received a lot of attention from scholars at home and abroad in recent years, such as transparent wood, wood sponge, straw plate, etc. Among them, biomass-based transparent wood (BBTW), as an emerging result of biomass materials research, have been widely studied for their light weight, light transmission, environmental protection, and high mechanical strength (Zhou and Wu, 2020). BBTW are mainly prepared renewable natural plant materials as raw materials and modifying their intrinsic structures or compounding them with other materials to achieve their light transmission and other functions.

At present, BBTW are still in the development and exploration stage, and the related research is focused on the preparation, but not yet put into actual production. The current researchs for BBTW are mainly focused on replacing architectural glass, luminescent materials, energy storage, battery substrates and other fields. This paper briefly discussed the research progress of BBTW, and summarizes the key technologies and potential application prospects of BBTW in replacing architectural glass.

## Preparation of BBTW and Light Transmission Principle

The development history of BBTW can be divided into three stages. 1) The first stage is the pre-exploration of the BBTW. In 1998, (Murayama et al., 1998) were the first to report the use of nanocellulose to prepare a translucent paper with high tensile strength. Following this innovation, many domestic and foreign scholars have initiated research on transparent paper. However, the process of preparing transparent paper has limitations such as high energy consumption and time consuming. 2) The second stage is the period of rapid development of BBTW. A new type of transparent wood (TW) was reported by Liangbing Hu’s team in 2016. As a result, the preparation of TW gradually became a research hotspot. 3) The third stage is the period of improvement and enhancement of the transparency of TW. In recent years, many scholars have also conducted research on the transparency of TW other than wood and proposed many methods to improve the performance of BBTW.

Natural wood contains lignin and other light-absorbing components, and the porous structure of the wood will scatter visible light, resulting in opacity of the wood, which can not meet the demand for lighting and can not be used as a building glass material. There are two main reasons for the opacity of wood: 1) The wood contains a large amount of light-absorbing substance that is lignin, which accounts for 20–30% of the total weight of wood; 2) The porosity of wood is as high as 30–80%, and a large number of pores have diameters larger than the wavelength of visible light (380–780 nm), which will cause severe light scattering. Therefore, by removing the lignin in the wood, it is possible to remove the chromogenic substances while retaining the wood skeleton structure. The pore structure in wood mainly includes a microcapillary system (mainly formed by the dynamic connection of tiny pores with a size below 10 nm in the fine running wall) and a large capillary system (mainly composed of conduits and screens). These capillary channels are connected to each other, so a transparent resin with a very high refractive index matching with the cellulose can be injected into it to fill the pores in the wood, thereby achieving transparency.

As shown in Table 1, BBTW were prepared by different preparation methods. The preparation of BBTW is divided into two main steps, preparation of delignified biobased templates (DBT) and impregnation of resins with matching refractive indices. The main methods for preparing DBT are acid, alkali, bioenzymatic and lignin modification methods. Sodium hypochlorite solution is usually used as a high frequency reagent for acid delignification. Sodium hypochlorite destroys the aromatic ring structure of lignin by oxidation and chlorination, thus achieving the effect of lignin removal. The most commonly used reagent for alkali delignification is a mixture of sodium hydroxide and sodium sulfite solution. In addition to the common treatment with sodium hydroxide solution, other alkaline solutions have also been used. Biological enzymatic delignification is a green delignification process, which degrades lignin by enzymes in order to achieve the purpose of delignification. By modifying the lignin, the chromophore groups are removed to achieve bleaching. After obtaining DBT, it is necessary to impregnate it with a resin matching its refractive index to obtain BBTW. The commonly used resins are: methyl methacrylate (MMA), epoxy resin, polyvinyl alcohol (PVA), and polyvinyl pyrrolidone (PVP). The choice of resins is not limited to the requirement of a high refractive index match with DBT, but is also increasingly focused on the environmental and physical properties of the resin itself. Current polymers are mainly petroleum-based products. One of the challenges is to replace fossil polymers in the manufacture of sustainable TW. Recently, Montanari et al. (2021) have successfully developed an environmentally friendly alternative: limonene acrylate, a monomer made from limonene. A substance extracted from orange juice is used to make a polymer that restores the strength of the delignification and allows light to pass through, producing BBTW.

**TABLE 1:** BBTW prepared by different methods.

Raw material	Delignin method	The transparency method	References
Balsa wood	NaClO_2_	PMMA	Chen et al. (2019)
Basswood	NaClO_2_	Epoxy resin	Mi et al. (2020b)
Basswood	Alkaline H_2_O_2_	Epoxy resin	Li et al. (2016)
Basswood	NaClO_2_	Mechanical thermal pressure	Zhu et al. (2018)
Balsa wood	Sodium subchlorite + alkaline H_2_O_2_	Sulcanol − Alene	Hoglund et al. (2020)
Balsa wood	NaClO_2_	PVA	Mi et al. (2020a)
Balsa wood	DES/Alkaline H_2_O_2_	PAA	Bi et al. (2018)
Balsa wood	PAA	Limonene acrylate	Montanari et al. (2021)

## Requirements and Limitations of Architectural Glass

### Requirements for Flat Glass in Architectural Glass

GB116114-2009 (China National Standardization Administration Committee 2010) provides relevant regulations on some properties of flat glass in building glass doors and windows. The standard specifies the minimum value of visible light transmission ratio of flat glass of different nominal thickness in architectural glass. The transmission ratio, also known as transparency, transmittance or transmission coefficient, is the ratio of transmitted luminous flux to incident luminous flux (Wang et al., 2020). As shown in Table 2, the minimum value of the visible transmittance ratio is 89% when the nominal thickness of flat glass is 2 mm, and as the nominal thickness increases, the minimum value of visible transmittance ratio is gradually reduced, and the minimum value of visible transmittance ratio should reach 67% when the engineering thickness of flat glass reaches 25 mm.

**TABLE 2:** Minimum visible light transmittance of colorless transparent flat glass (Wang et al., 2020).

Nominal thickness/mm	Minimum value of visible light transmission ratio/%
2	89
3	88
4	87
5	86
6	85
8	83
10	81
12	79
15	76
19	72
22	69
25	67

### Requirements for Fireproof Glass in Architectural Glass

GB15763 ″Safety Glass for Buildings” (China National Standardization Administration Committee 2009a) contains the following four types of glass: fireproof glass, tempered glass, laminated glass and homogeneous tempered glass. As shown in Table 3, the fire resistance performance is specified in GB15763.1-2009 “Fireproof Glass” (China National Standardization Administration Committee 2009b).

**TABLE 3:** Fire-resistant properties of fireproof glass (China National Standardization Administration Committee 2009b).

Classification name	Fire resistance limit grade (h)	Fire-resistant performance requirements
Heat-insulating fireproof glass (Class A)	3.00	Fire resistance and heat insulation time≥3.00 h , and fire resistance integrity time≥3.00 h
	2.00	Fire resistance and heat insulation time≥2.00 h , and fire resistance integrity time≥2.00 h
	1.50	Fire resistance and heat insulation time≥1.50 h , and fire resistance integrity time≥1.50 h
	1.00	Fire resistance and heat insulation time≥1.00 h , and fire resistance integrity time≥1.00 h
	0.50	Fire resistance and heat insulation time≥0.50 h , and fire resistance integrity time≥0.50 h

And the standard also specifies the impact resistance of single fire glass. Composite fire glass is not destroyed means that the glass after the test to meet one of the following conditions: first, the glass is not broken; second, the glass is broken but the steel ball did not penetrate the specimen (China National Standardization Administration Committee 2009a).

### Requirements of Thermal Insulation Glass for Building

JC/T2304-2015 Technical conditions of thermal insulation glass for building (Ministry of Industry and Information Technology of the People’s Republic of China 2015) stipulates that there are three types of thermal insulation glass for building, which are thermal insulation type BW, thermal insulation type GR, thermal insulation type BG. As shown in Table 4, for the performance of three different types of thermal insulation glass, this standard stipulates its conduction coefficient (K), light to heat ratio (LSG), solar radiation spectrum transmittance ratio (GIR).

**TABLE 4:** Technical conditions for insulating glass (Ministry of Industry and Information Technology of the People’s Republic of China 2015).

Types	Levels	Technical conditions		
		K(W/m^2^·K)	LSG	GIR
Thermal insulation type BW	BW1	≤1.8	≥1.0	—
	BW2	≤1.5	≥1.0	—
	BW3	≤1.0	≥1.0	—
thermal insulation type GR	GR1	≤2.5	≥1.0	≤0.40
	GR2	≤2.2	≥1.5	≤0.20
	GR3	≤2.0	≥1.9	≤0.05
thermal insulation type BG	BG1	≤2.0	≥1.0	≤0.40
	BG2	≤1.8	≥1.5	≤0.20
	BG3	≤1.5	≥1.9	≤0.05

### Limitations of Architectural Glass

Architectural glass have many advantages, such as high transmittance, strong sense of fashion, good permeability, rich modeling, wide application, etc. However, there are some limitations of glass windows and doors, specifically in the following aspects.

First, they can produce discomfort glare to threaten public safety. Glare is the visual discomfort caused by the extreme contrast of brightness. The human eye can not adapt to this unsuitable brightness distribution, which may give people a sense of nausea and discomfort. In cities, many buildings are decorated with large-format glass windows on their facades, although when the light is strong, the glare from glass windows may be directed to the eyes of surrounding pedestrians and vehicle drivers, causing visual fatigue and even causing traffic accidents.

Second, the high brittleness of glass will make the cracks in glass very easy to expand by the fact of loosen in the crystal structure due to the less dense of crystallization with coarse grains. Therefore, in the handling, installation, and use process of glass, when accidental colliding happens, it is easy to produce fragments, threatening people’s safety.

Third, there are drawbacks in the production process of flat glass industry. The SO_2_, NO_x_ emission reduction pressure is still greater, and the energy saving and emission reduction technology needs to be improved (Zhao et al., 2017). As China’s flat glass environmental protection facilities put into use for a relatively short period of time, the periodically “change fire” operation of kiln in the production process always results in unstable concentration of exhaust gas emissions.

## Advantages and Limitations of BBTW as Alternative Glass Materials

### Advantages of BBTW as Alternative Glass Materials

#### Optical Properties of BBTW Meet the Requirements of Architectural Glass

**FIGURE 1:**
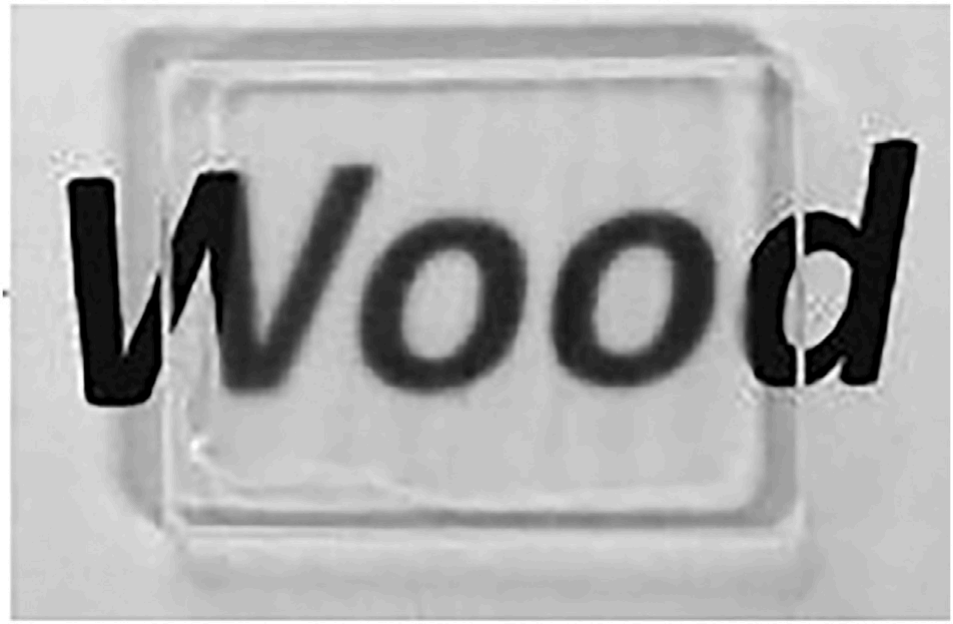
Macroscopic observation diagram of TW (Zhu et al., 2016).

BBTW should not only meet the basic requirements of glass when used in place of glass for architectural glass, as shown in Figure 1 (blow), Zhu et al., 2016 prepared 2 mm TW with a transparency of up to 90%, and this study met the requirements of GB116114-2009 on the visible light transmission ratio of flat glass. Therefore, through certain means and techniques, the light transmission ratio of BBTW can reach the requirements of architectural glass.

**FIGURE 2:**
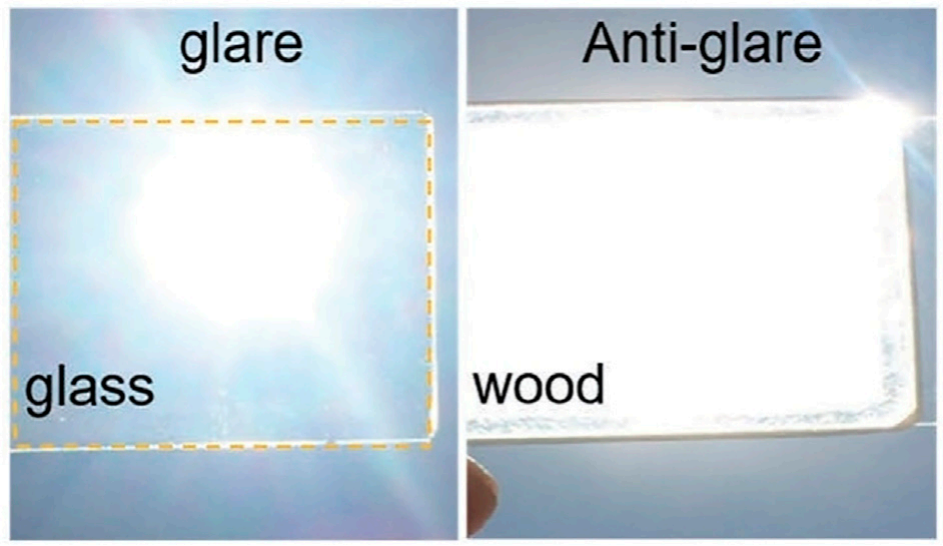
Glare contrast between glass and TW (Li et al., 2016).

BBTW also need to compensate on the disadvantages and shortcomings of glass. As shown in Figure 2 (blow), Li et al., 2016 compared glass and TW against the Sun; glass produces glare due to its extremely uneven brightness, and glare interferes with the clarity of the visual image, while TW has a more uniform and comfortable light due to its uniform texture.

**FIGURE 3:**
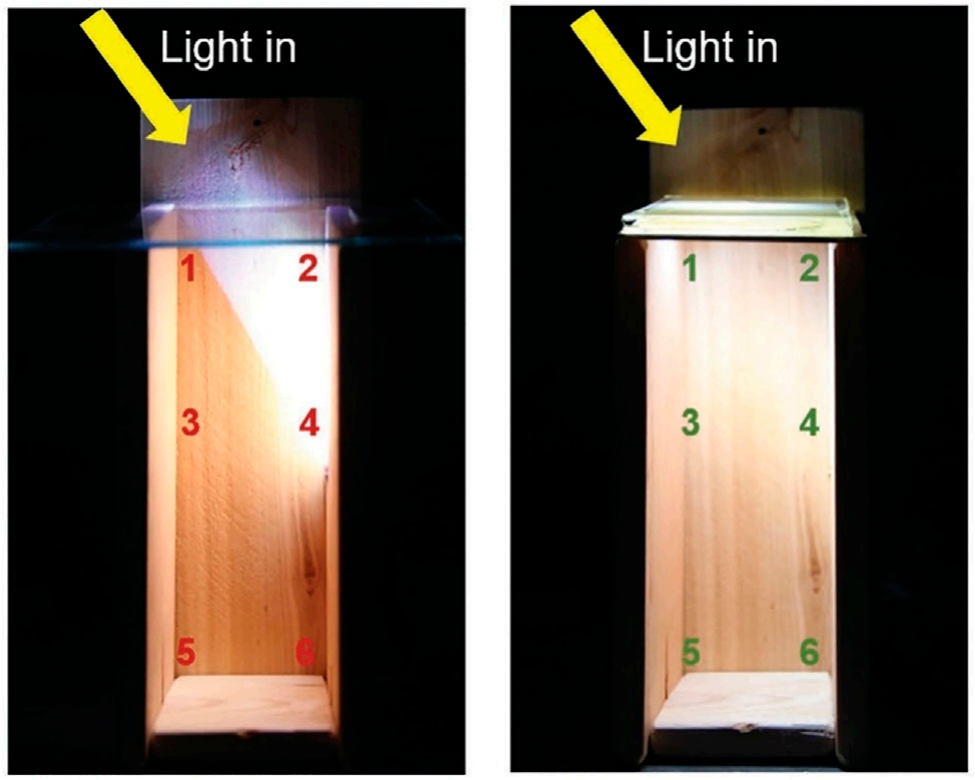
Light contrast between glass and TW (Li et al., 2016).

As shown in Figure 3 (blow), Li et al., 2016 simulated the skylight of a building with glass and TW, and chose six different locations respectively. With glass as the skylight material, the light intensity in the brightest part of the house was 35 times greater than that in the darkest part, and the light was clearly uneven. On the contrary, the difference in light intensity between the brightest and the darkest parts of the house was only 2.3 times when TW was used as the skylight material.

#### BBTW Is Safer and has Excellent Mechanical Properties

**FIGURE 4:**
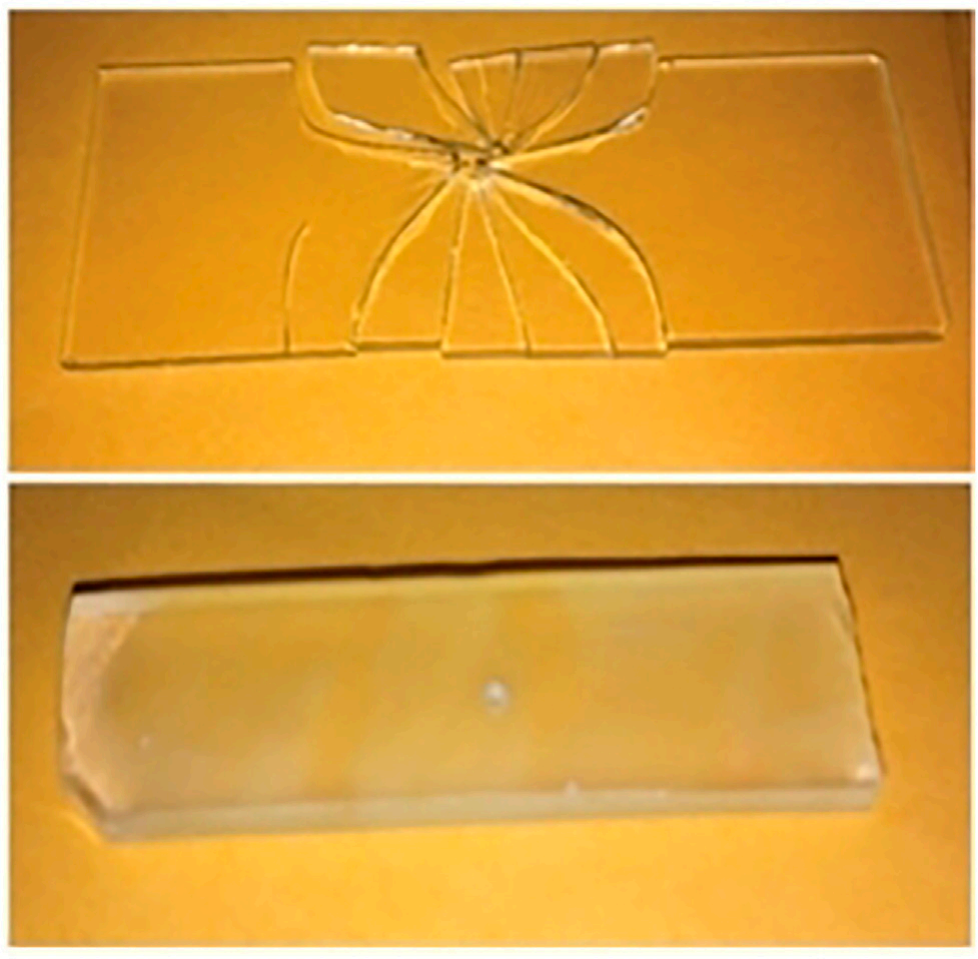
Brittle contrast between glass and TW (Li et al., 2016) (Upper right: glass; Lower right: TW).

As shown in Figure 4 (blow), glass immediately shatters into sharp fragments when struck by a falling sharp object. In contrast, even after a sudden impact, TW only bends and splits, rather than shattering into multiple sharp fragments. Therefore, compared to glass materials, BBTW do not have safety hazards caused by shattering. Also, BBTW exhibit excellent mechanical properties. For example, the tensile strength of transparent bamboo (TB) prepared by Wang et al. (2018) reached 92 MPa; the maximum tensile strength of TW prepared by Wu et al. (2019) reached 165.1 ± 1.5 MPa.

#### Green Production Process

Green process technology is in the key part of the whole process of product life cycle and is the key process to make a green product. In the production process of flat glass, the emission of pollutants and the loss of energy are very huge (Zhao et al., 2019). In contrast, the process of biomass material transparency is free from the emission of toxic and harmful gases. For example, the epoxy resin as impregnating material is free from irritating odor. Recently, Montanari et al. (2021) have successfully developed an environmentally friendly alternative: limonene acrylate, a monomer made from limonene. Besides, the biomass used as raw materials have the advantages of being very widely sourced, green and renewable, which are in line with the trend of sustainable development.

#### Higher Aesthetic Value

**FIGURE 5:**
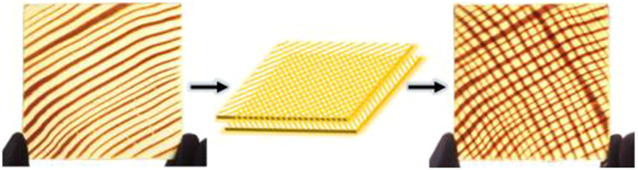
Design of TW (Mi et al., 2020a).

Wood materials not only have practical value but also have high aesthetic value in terms of texture, and the patterns formed by the different arrangement of cell distribution are variable (Yin and Liu, 2020). Compared to glass, most wood materials have a unique texture that is maintained after the transparency treatment, and more types of patterns can be achieved by superimposing two layers of transparent wood. As shown in Figure 5 (blow), Ruiyu Mi et al. (2020a) designed various lattice patterns by stacking two layers of TW rotated at opposite angles. In addition, using this universal manufacturing method, the BBTW developed from wood with natural texture can increase the aesthetic value of the building.

#### Good Thermal Insulation Properties

Zhang Y. et al. (2018) compared the radial thermal conductivity of glass, TW, transparent wood fiber (TWF) and unmodified wood (UW), and the thermal conductivity of glass, TW, TWF and UW were 1.033, 0.164, 0.178 and 0.124 W m^−1^K ^−1^. The thermal conductivity of TWF and TW was significantly lower than that of glass and slightly higher than that of UW. As shown in Table 5, the thermal conductivity of the transparent materials prepared from different biomass materials were all significantly lower, which fully satisfied the requirements for thermal conductivity of thermal insulation glass in the above Technical Conditions for Thermal Insulation Glass for Buildings. Wang et al. (2018) found that transparent wood has better thermal insulation performance. Under the condition of an external temperature of 4°C, the indoor temperature of a simulated house using transparent wood dropped from 35°C to 20.1°C. At the same time, the simulation using glass The indoor temperature of the house dropped from 35.1°C to 9.1°C. Applying BBTW to replace architectural glass will help slow down internal temperature fluctuations and reduce energy consumption.

**TABLE 5:** Comparison of thermal conductivity of transparent materials prepared from different materials.

Material type	Polymer	Radial thermal conductivity (W m^−1^K^−1^)	References
Basswood	Epoxy resin	0.32	Li et al. (2016)
Douglas-fir	Epoxy resin	0.24	Mi et al. (2020b)
Poplar	PMMA	0.164	Zhang Yaoli et al. (2018)
Balsa	PMMA	0.23	Li et al. (2019)
Pine wood	Epoxy resin	0.2	Zhang et al. (2020)

### Challenges for BBTW to Replace Architectural Glass

#### Increasing the Width

**FIGURE 6:**
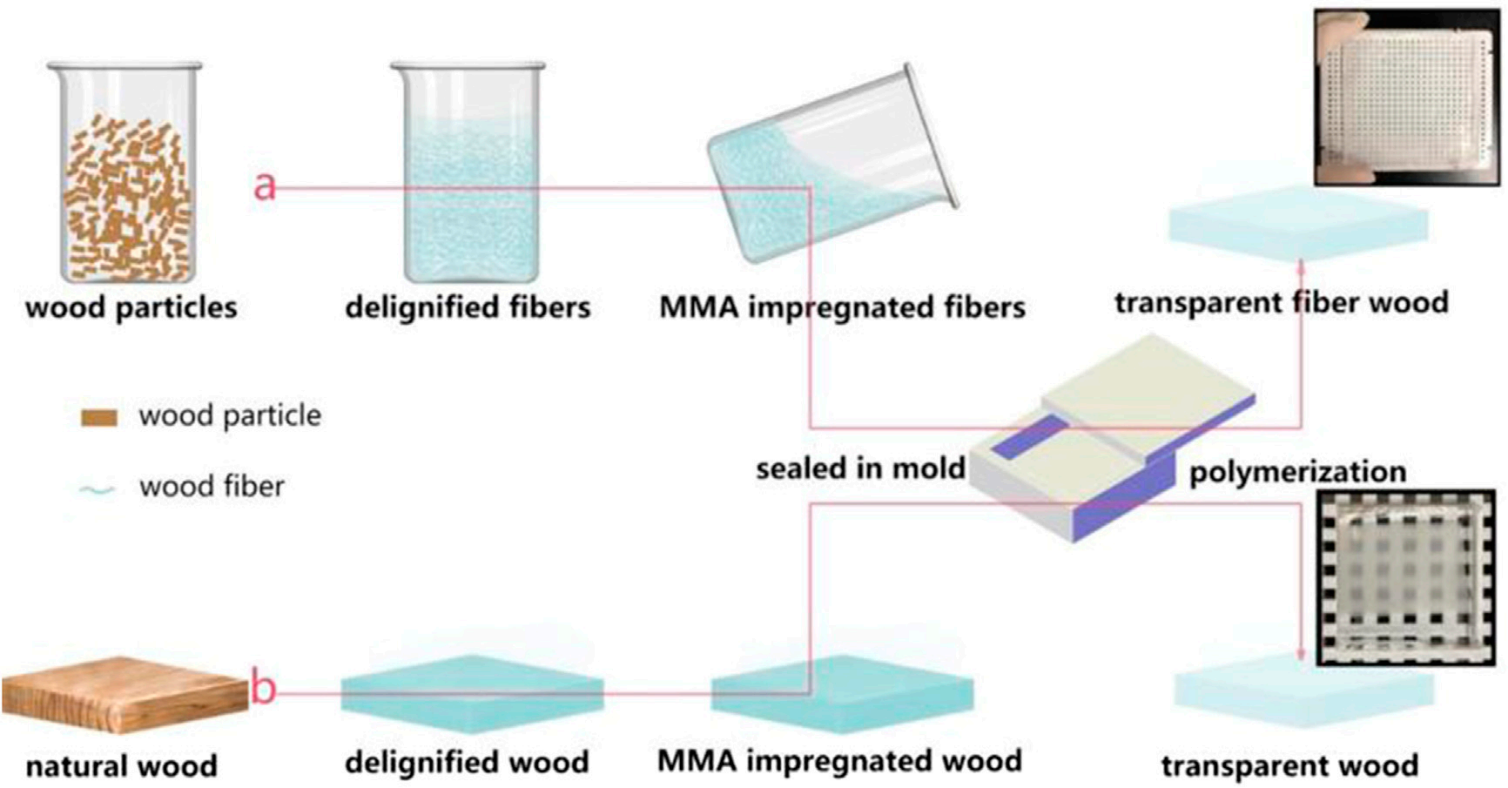
Comparison and of the whole wood material transparency and wood fiber transparency process (Wang et al., 2018).

At present, the research on the BBTW is still in the laboratory stage, and the size of the prepared BBTW is limited. If BBTW are to be used in architectural glass, the first thing to consider is to increase the width. Since it takes more time to delignify a large size biomass material (e.g. a whole piece of wood or a whole section of bamboo) and it is difficult to fill it completely with resin when impregnating it, it is inevitably more difficult to prepare a whole biomass material directly for direct transparent processing. The whole biomass material can be processed into fibers and the biomass fibers can be transparently treated, which can reduce the preparation difficulty and at the same time can prepare BBTW of arbitrary size to adapt to the dimensional changes of architectural glass in different scenarios. As could be seen from Figure 6 (blow), Wang xuan et al. (2018) prepared TW by this method not only with the size of 300 mm × 300 mm × 10 mm and 68% light transmission, but also with high preparation efficiency, which is suitable for mass production.

#### Improve Light Transmission

**FIGURE 7:**
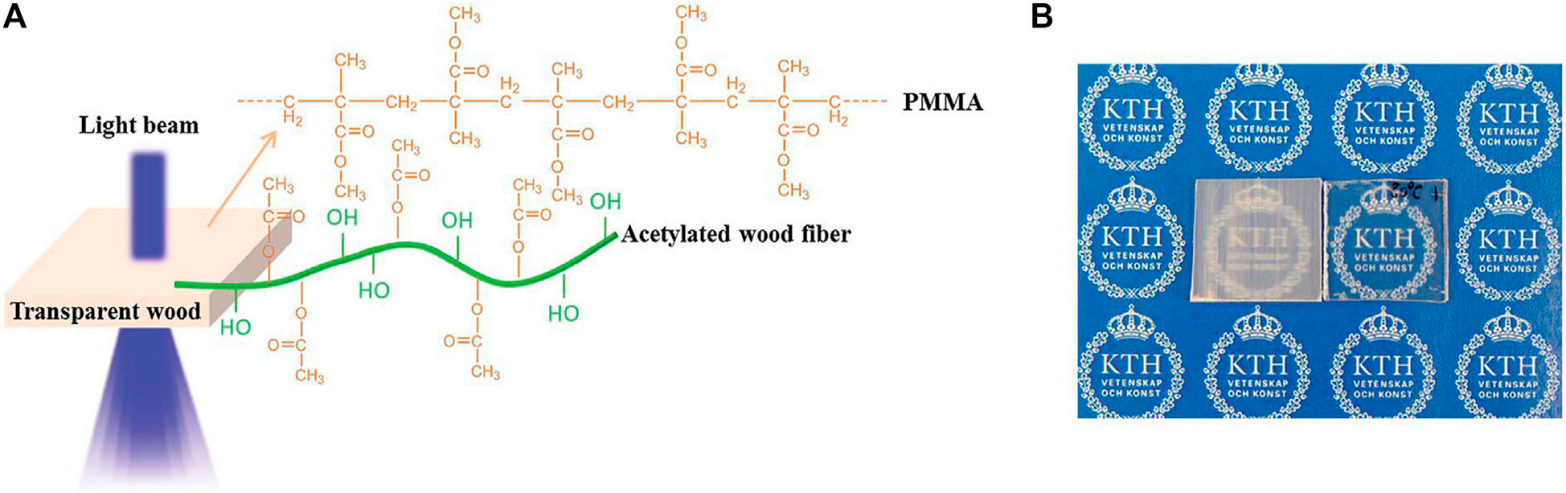
**(A)** Schematic representation showing the structure of the modifified highly transparent wood. The wood template was acetylated to have better compatibility with PMMA. **(B)** Photograph shows the non-acetylated transparent wood (left) and acetylated transparent wood (right). (Li et at. 2018b).

Although the light transmittance of existing BBTW is being improved, there is still a gap between them and the standard of flat glass used in architectural glass. Therefore, it is crucial to improve the light transmission of BBTW. The reason why the light transmission of BBTW can not reach the standard of flat glass is due to the debonding phenomenon at the interface of various parts of the composite material, i.e., cracks at the interface between the polymer and the cell wall of the biomass material, which leads to light scattering or reflection, thus reducing the light transmission of BBTW. This limitation can be improved by acetylation treatment. Li et al. (2018a) prepared high transmittance BBTW with a thickness of 1.5 mm and 93% light transmission by acetylation treatment of TW (see Figure 7B blow). As shown in Figure 7A, acetylation treatment is the reaction of introducing acetyl CH3CO- on the nitrogen, oxygen, and carbon atoms in the molecules of organic compounds. The acetylation treatment increases the compatibility of the cell wall of the biomass material with the polymer, thus increasing the light transmission.

#### Improve Flame Retardancy

Flame retardancy is also a key performance indispensable for architectural glass. Improving the flame retardancy of BBTW can be achieved by adding flame retardant materials for fire protection purposes. For example, polyimide can be added to biomass materials in the process of transparency. Polyimide (PI) is a polymer with imide ring on the main chain, which has the advantages of high thermal stability, excellent mechanical strength, radiation resistance, non-toxicity, and self-extinguishing, etc. Chen et al. (2020b) demonstrated that after adding PI, BBTW maintain good self-extinguishing performance within 2 s after leaving the fire source, the reason is that the thermal decomposition temperature of PI is generally 500°C, and the decomposition products are mainly non-polluting, non-combustible nitrogen-containing gases, which can both dilute the concentration of combustible gases and achieve the pursuit of flame retardancy.

#### Smart Dimming

If BBTW are used to replace architectural glass, they should not only meet some basic performance requirements, but also conform to the current concept and development trend of smart home. For example, it needs to have the function of adjustable haze, so that it can meet the role of privacy protection in certain scenarios and can be switched for different needs and different scenarios. Qiu et al. (2020) prepared TW with thermally reversible optical properties by infiltrating copolymers of monomer styrene (St), butyl acrylate (BA), and octadecene (ODE) into the treated wood. The introduction of ODE chains leads to TW with repeatable optical properties. With increasing temperature, TW can change repeatedly from lower visibility (transmittance: 23.7%, haze: 8.3%) to transparency (transmittance: 74.9%, haze: 36%), with the opposite change occurring during cooling. This technique promotes the potential application of BBTW as a material for smart windows and doors.

#### Self-Cleaning Properties

In addition to some basic performance, BBTW can also add some other functions to extend the life cycle of BBTW. For example, self-cleaning function is very important. If architectural glass is to be self-cleaning, it needs to be coated with hydrophobic paint on the surface of the glass. Hydrophobic paint is sprayed on the glass surface, and a durable nano protective film can be formed within a few seconds, which plays a role of self-cleaning and care. The unique nano-structured protective film imitates the super hydrophobic and self-cleaning function of the natural lotus leaf. The water droplets on the coating surface slide quickly as if falling on the lotus leaf, and the coating surface is kept clean. However, BBTW itself has self-cleaning properties. For example, the transparent luminous wood prepared by Salhah et al. (2021) has superhydrophobic activity, and the water contact angle of the transparent luminous wood is increased from 151.9° to 162.8°, which has extremely superior self-cleaning characteristics.

#### UV and IR Shielding Performance

Ordinary architectural glass does not have the ability to resist ultraviolet radiation, which will accelerate the aging of human skin. Antimony-doped tin oxide (ATO) particles have excellent ultraviolet and near-infrared shielding properties, and are usually used in shielding glass. Qiu et al. (2019) used modified antimony-doped tin oxide (ATO) Preparation of ATO/transparent wood. When the addition amount of ATO is 0.7% (wt), the UV shielding effect of ATO/transparent wood is as high as 80%. When applied to windows, it can reduce the damage of ultraviolet rays to human skin.

## Future Prospects of BBTW

### General Windows and Doors

A well-lit environment can bring more comfortable living experience, while on the contrary, a poorly lit environment can have a negative impact on the human body and mind. Compared with glass, BBTW could meet the conditions of no glare and uniform light exposure, which are important for lighting in houses. BBTW can be used so that artificial lighting can be partially replaced by sunlight to reduce energy (Li et al., 2018b). This initiative is in line with the current concept of green development and sustainability. BBTW are also highly non-toxic and can ensure a more uniform and soft transmission of light, thus protecting interior privacy. However, its application to ordinary windows and doors also needs to be continuously increased and improved light transmission rate, so as to meet the conditions of use of flat glass. Attention should also be paid to the requirement of flame retardancy by adding thermal insulation and fire retardant reagents. The application of BBTW as an alternative to colorless transparent flat glass in architectural glass is promising.

### Thermal Insulated Windows and Doors

In addition to ordinary flat glass, BBTW can be used for thermal insulated windows and doors applications. Li et al. (2018a) prepared TW for thermal insulated windows in buildings. Compared to glass windows, transparent wood windows have less temperature change under continuous solar radiation, which contributes to energy saving and environmental protection. The use of transparent cross-laminated wood can be considered to enhance the stability of the structure, but still sufficient thermal resistance (V Karl’a 2019.) Energy-efficient buildings with BBTW as insulated windows and doors consume less energy, which not only significantly reduces electricity consumption, but also promotes natural and comfortable interior lighting.

### Intelligent Doors and Windows

With the continuous improvement of people’s living standards, the concept of smart home has come into being, and smart windows and doors are an indispensable part of it (Cao et al., 2018). The application of BBTW in smart windows and doors has the following target directions. First, when the ambient temperature of biomass transparent windows and doors is adjusted, the light transmittance and haze of the windows and doors will change accordingly. For example, when reaching a higher temperature, the light transmittance of windows and doors decreases and the haze increases, which can protect the privacy of users, and when the temperature decreases, the light transmittance of windows and doors increases, the haze decreases and the lighting is good. Secondly, BBTW can be used as a smart door and window to protect against ultraviolet rays to protect the indoor environment and protect the skin health of the occupants, and delay skin aging.

### Replacement of Glass Curtain Wall

Building glass curtain wall uses a lot of glass materials, a certain extent that can meet the role of beautifying the appearance of the building, but its disadvantages can not be underestimated. Glass curtain wall will not only cause light pollution, but also security risks. Due to the long-term adverse effects of the natural environment, the structure of the glass curtain wall is easy to aging, resulting in the fall of the glass curtain wall. BBTW not only has aesthetic value, but also has unique texture and color. At the same time, the texture of BBTW is even, even if it is impacted, it will not break into sharp pieces. Due to its excellent optical performance, fire and heat insulation performance and high safety factor, BBTW has a broad prospect in replacing building glass curtain wall.

## Conclusion

Compared with the steel and concrete structures commonly used in modern architecture, house components made of wood materials can make occupants closer to nature and more relaxed. At the same time, BBTW meet the requirements of good lighting conditions as well as flame retardancy, heat insulation and safety, and have the advantages of very wide material sources, green and renewable, which are in line with the trend of sustainable development. However, due to issues such as process, time, cost and safety, there is still a certain gap between transparent wood and actual large-scale application. Improving the transparency of large-size materials and finding industrialized production methods are the hotspots and difficulties in the current research on transparent wood. Judging from the current research, two problems need to be solved 1) to achieve large-size and high light transmittance biomass-based transparent wood; 2) to find new ideas for preparation, adding more functions, intelligence, composite and other related content, Such as phase change energy storage, electric heating, flame retardant, self-cleaning, smart dimming, decoration, etc. The application of biomass-based transparent wood in architectural glass need gain more experience that would be replicable and scalable in the future with an aim to achieve large-scale and batch production and achieve sustainable development.
